# Association of derived patterns of musculoskeletal disorders with psychological problems: a latent class analysis

**DOI:** 10.1186/s12199-019-0784-x

**Published:** 2019-05-15

**Authors:** Maryam Yazdi, Sara Karimi Zeverdegani, Amir Hossein MollaAghaBabaee

**Affiliations:** 1 Department of Biostatistics and Epidemiology, School of Health, Isfahan Endocrine & Metabolism Research Center, School of Medicine, Isfahan University of Medical Sciences, Isfahan, Iran; 2 Department of Occupational Health Engineering, School of Health, Isfahan University of Medical Sciences, Isfahan, Iran; 3 Isfahan Provincial Health Center, Isfahan University of Medical Sciences, Isfahan, Iran

**Keywords:** Anxiety, Depression, Latent class analysis, Musculoskeletal disorders, Stress

## Abstract

**Background:**

Musculoskeletal symptoms often occur in more than one anatomical site. The present study aimed to define specific patterns of multisite musculoskeletal disorders and examine how these patterns are related to common psychological problems.

**Methods:**

Using the data from an interview-based health survey of 358 samples of the industrial manufacturing male employees, we derived major patterns of musculoskeletal complaints using latent class analysis and investigated its association with psychological problems score extracted from depression, anxiety, and stress measured by Depression/Anxiety/Stress Scale (DASS-21). Musculoskeletal disorders were assessed by Nordic Musculoskeletal Questionnaire (NMQ). The statistical analysis was carried out by Mplus 8.

**Results:**

Complaints in the lower back (42.1%) and neck (30.7%) had the highest prevalence, and in the hip (15.0%) and ankle (12.2%) the lowest. Three major patterns of musculoskeletal disorders were extracted using latent class analysis. Class 1 (12.9%) was characterized by a high rate of complaints in upper musculoskeletal sites, such as the neck, shoulder, and joints; class 2 (38.2%) was identified by a higher rate of complaints in the lower and upper back; and class 3 (48.9%) was marked by low rates of complaints in all musculoskeletal sites. After adjustment for confounding variables and specifying class 3 as the reference, it turned out that there was a statistically significant association between the psychological problems score and the chance of being in class 1 (OR = 2.47, 95% CI 1.66–3.68), but not a significant association with the chance of being in class 2 (OR = 1.51, 95% CI 0.83–2.72).

**Conclusion:**

Musculoskeletal disorders can be summarized in the latent class-derived patterns in the adult study population and provide additional prognostics. Common psychological problems are significantly associated with the type of musculoskeletal disorder patterns. The findings in this study could be useful for dealing with prevention and treatment programs.

## Introduction

Musculoskeletal disorders (MSDs) are a common problem in the general population [1]. MSDs are defined as a feeling of discomfort, difficulty, or pain in the musculoskeletal system (joints, muscles, tendons) or soft tissues of the body. Musculoskeletal symptoms and disorders encompass pain and other symptoms at specific anatomical sites. Chronic MSDs affects between 11% and 50% of the general population [2, 3]. MSDs are also considered as the most important cause of occupational injury and disability in the world and the main cause of absenteeism [4, 5].

It is, additionally, remarkable that the majority of available researches have evaluated MSDs as site specific, but recent studies have emphasized on a multisite investigation of MSDs in the working [6–10] and general population [11–14], indicating the moderate prevalence of symptoms strictly confined to a specific anatomical site (estimated prevalence of 15–30% in different studies) and the noticeable prevalence of multisite symptoms (estimated prevalence of one third and two thirds in the general and working population, respectively). Furthermore, it was clearly described that although the involvement of several sites in the same region was very common, involvement of several sites located in separate regions was also common [11].

Several studies have described the profiles of multisite MSDs [7–10, 13] using 2 by 2 combinations or number of sites involved with the impairment. In the meanwhile, few studies have analyzed major patterns of MSDs and classified their populations through latent variable modeling approaches such as latent class analysis (LCA) [14–17]. LCA is based on structural equation modeling, which allows the identification of latent groups based on a set of observed variables. Indeed, it can be useful to point out how people are clustered according to the patterns of MSDs as well as how the association between these groups and some external factors such as psychological problems are identified [18]. Clinical and epidemiological studies indicate musculoskeletal symptoms could be the manifestations of mental illnesses in the form of physical symptoms [19]. Several studies have reported a significant association between psychological disorders and pain in sites of back, neck, head, joints, or face [20–22]. In a systematic review, it was indicated that depressive symptoms are associated with higher levels of pain intensity, more functional limitation and disability, and worse prognosis [23]. However, there are not any papers investigating the association of major patterns of MSDs and psychological problems.

The primary aim of the present study was to derive major patterns of MSDs and classify the study population into more homogenous subgroups using LCA. Secondly, the study examined whether the extracted MSDs patterns were associated with an external factor, such as psychological problems score (a combined measure extracted from self-reported anxiety, depression, and stress scores), controlling for demographic, job, and lifestyle-related variables.

## Methods

### Study design and subjects

This cross-sectional study was conducted among 1200 employees in two administrative and manufacturing sections of a metal industry in 2014 in Iran. Due to a low number of female employees (3 individuals) the study results were restricted to males. Individuals with a history of surgery in the spine (7 individuals) and musculoskeletal disorders injuries (43 individuals) were excluded from the study. Participants were sampled in a convenience-sampling scheme along with stratified sampling for administrative and manufacturing sections. In fact, the sample size in each stratum was proportional to its size. Finally, 358 individuals met the study criteria. The study protocol was clarified for all participants and a signed written informed consent was obtained from them.

### Measurements

#### Musculoskeletal disorders

MSDs were evaluated using Translated Nordic Musculoskeletal Questionnaire (NMQ). The Nordic questionnaire consists of structured and forced multiple choice questions, and can be used as a self-administered questionnaire or an interview [24]*.* The participants were asked about having any trouble regarding pain or discomfort during the last 12 months/7 days in any these sites (the neck, shoulders, upper back, lower back, elbow, wrists, knees, ankles, and legs). Each person could respond “yes” or “no” to any of the complaints and more than one confirmatory answer was allowed. [25]. The validity and test-retest reliability of the translated version of NMQ were investigated in [26, 27]. Pearson correlation coefficients were found between 0.88 and 1.00 for different musculoskeletal sites in [27].

#### Psychological problems score

Psychological problems score was extracted from stress, anxiety, and depression using factor analysis. Stress, anxiety, and depression were assessed by Depression/Anxiety/Stress Scale (DASS)-21 questionnaire [28]. The DASS-21 is the short form of DASS-42, containing 21 items (7 per scale). Actually, it assesses three constructs: depression, anxiety, and stress [29]. The respondents to the questions were asked to score every item on a scale from 0 (did not apply to me at all) to 3 (applied to me very much). Moreover, the sum scores were computed by adding up the scores on the items per subscale and were doubled to be equivalent to the longer DASS-42 version. Sum scores for each of the subscales could range between 0 and 42. Depression Scores ≥ 21, anxiety scores ≥ 15, and stress scores ≥ 26 were labeled as “high” or “severe” [30]. Validity and reliability of the Persian version of DASS-21 were investigated for the Iranian population [31]. In the current study, internal consistency of the DASS-21 subscales was found to be acceptable, with Cronbach’s alphas of 0.91, 0.87, and 0.90 for depression, anxiety, and stress, respectively.

#### Covariates

The demographic characteristics and some lifestyle- and job-related variables of the subjects were collected by a questionnaire. The questionnaire includes several questions such as age, gender, marital status, body posture when working (sitting, standing, siting, and standing), duration of work hours, job section (manufacturing, administrative), having regular physical activity, and smoking currently***.***

### Statistical analysis

First, we performed a descriptive analysis. The prevalence of MSDs in the different anatomical sites was tabulated by age groups. Second, for reported MSDs, we used latent class analysis (LCA) to identify latent class-derived patterns of MSDs. LCA was built on the assumption that the association between MSDs could be explained by an underlying variable with an unknown number of classes known as latent classes. The optimal number of classes were determined using goodness of-fit criteria, which, by definition, must be as small as possible [32]. We used Akaike’s Information Criterion (AIC) and Bayes Information Criterion (BIC), recommended to determine the optimal number of classes [33]. After selecting the best model, we assigned each participant to one class according to his highest computed probability of membership. Basically, average posterior probabilities above 70% indicate optimal fit [32].

The association between the employees’ psychological problems score and MSDs derived patterns was also investigated in a posterior analysis using multinomial logistic regression in which psychological problems score were extracted from combining stress, depression, and anxiety scores using factor analysis. The results of regression models were adjusted for the impact of confounding variables, including demographic, job, and lifestyle-related variables. All analyses were performed using Mplus version 8 [34].

## Results

### Descriptive analysis

Of 358 employees participated in the study, 317 (88.5%) were married and the mean age was 40.68 ± 8.64 years. Work-hour duration for 98 (27.4%) participants was more than 8 h. Ninety-seven (27.1%) participants worked at administrative section and 261(72.9%) at manufacturing section. Forty (11.2%) participants had a sitting posture while working, 43 (12.0%) standing, and 275(76.8%) sitting and standing. A regular physical activity was reported by 145 (40.5%) of participants, and experience of smoking by 125(34.9%). Stress, anxiety, and depression were discovered to be high in 2.8%, 17.8%, and 2.8% of participants, respectively.

The prevalence of site-specific MSDs has been reported in Table 1. While complaints in lower back and neck had the most prevalence, pain in hip and ankle had the lowest among the total sample. Disparity of MSDs prevalence by age was clear in the underlying sample, the older age group (≥ 45) had higher rates of MSDs in all sites, and it was statistically significant in the lower back, neck, shoulder, knee, hip, and ankle (*p* value< 0.001).

**Table 1:** Prevalence of the site-specific MSDs at aged 18 year and older

	Age groups			
	18–44	≥ 45	Total	*p* value^b^
	241	117	358	
Lower back	82 (34.0)^a^	68 (58.1)	150 (41.9)	< 0.001
Neck	53 (22.0)	56 (47.9)	109 (30.4)	< 0.001
Upper back	52 (21.6)	36 (30.8)	88 (24.6)	0.058
Shoulder	45 (18.7)	38 (32.5)	83 (23.2)	0.004
Knee	42 (17.4)	41 (35.0)	83 (23.2)	< 0.001
Wrist	47 (19.5)	29 (24.8)	76 (21.2)	0.251
Elbow	34 (14.1)	25 (21.4)	59 (16.5)	0.082
Hip	28 (11.6)	26 (22.2)	54 (15.1)	0.009
Ankle	21 (8.7)	22 (18.8)	43 (12.0)	0.006

^a^number (%), ^b^resulted from Pearson chi-square test

### Latent class analysis

The MSDs major patterns and latent structure or unobserved heterogeneity of the study population were recognized using LCA. Table 2 shows fit statistics of LCA model for different numbers of classes. The model with three classes provided the best fit with the smallest BIC (BIC = 3285.12). Class counts and proportions based on the estimated posterior probabilities were as follows: there were 46 (12.9%) participants in class 1, 137 (38.2%) participants in class 2, and 175 (48.9%) in class 3. The average posterior probabilities for all three classes exceeded 0.7 (0.89 for class 1, 0.83 for class 2, and 0.90 for class 3), implying accurate classification of the participants to the correct class.

**Table 2:** Fit statistics for latent class analyses

Number of latent classes	Number of parameters estimated	LL	AIC	BIC
1	9	− 1695.77	3409.54	3444.54
2	19	− 1599.64	3237.29	3311.18
3	29	− 1557.17	3172.35	3285.12
4	39	− 1529.99	3137.98	3289.65
5	49	− 1510.61	3119.21	3309.77

*LL* Log likelihood, *BIC* Bayes Information Criterion, *AIC* Akaike’s Information Criterion

**Fig. 1:**
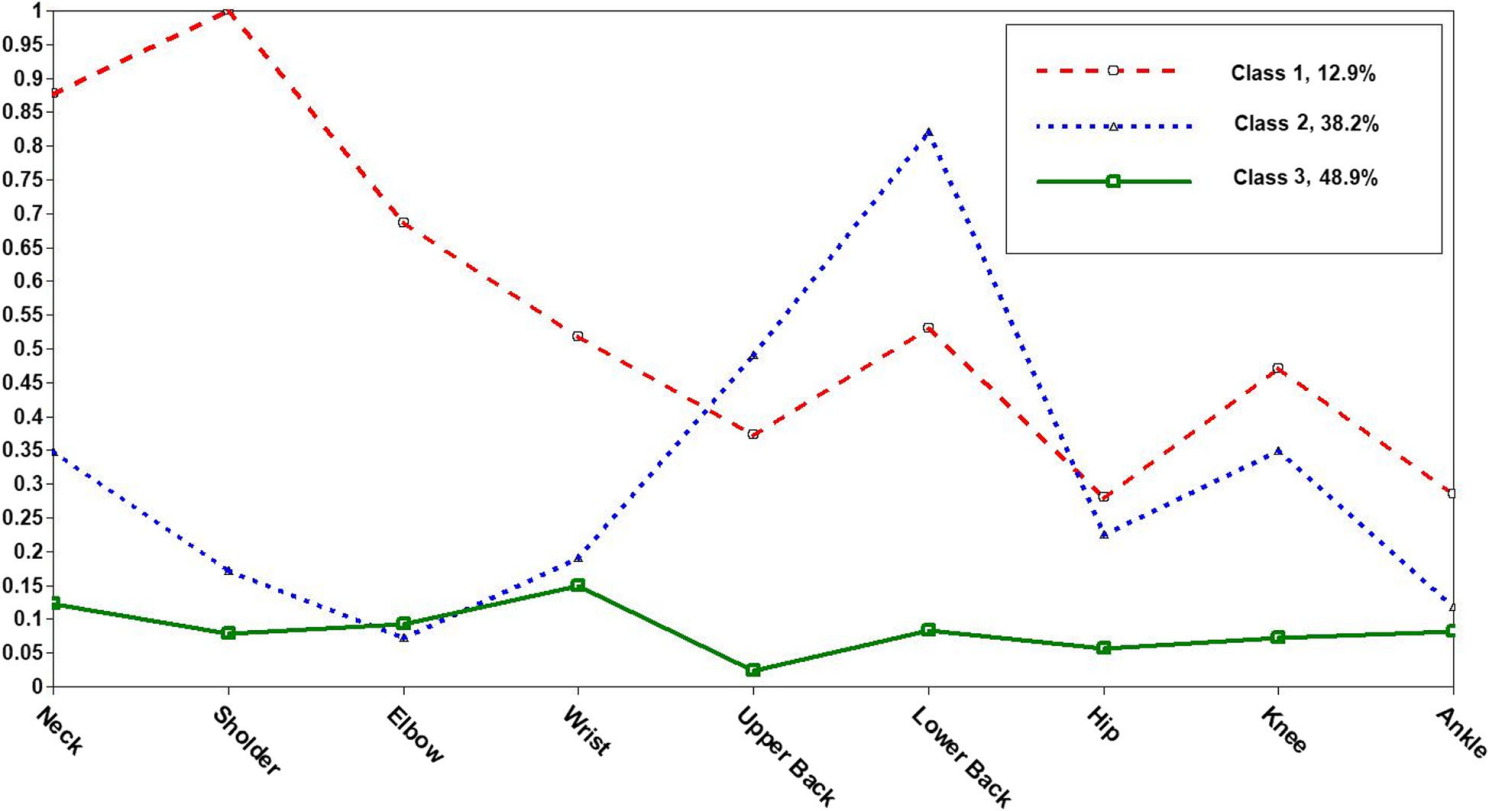
Self-reported frequency of MSDs stratified by latent class-derived patterns

Figure 1 illustrates sample proportions of reported MSDs across three identified classes. Class 1 was characterized by individuals with high probabilities of MSDs in upper organs (neck, shoulder) and joints as well as moderate probability of complaint in back area. Class 2 was marked by individuals with high probability of MSDs in the lower and upper back. And, class 3 accounted for almost half of the sample (48.9%), included individuals with near-zero probabilities of MSDs across all sites.

### Posterior analysis: association of MSDs patterns with psychological problems score

Using exploratory factor analysis, psychological problems score was extracted as a latent construct from combining three observed measures of depression, anxiety, and stress with factor loadings equal to 0.89, 0.90, and 0.88, respectively. The factor was extracted based on correlation matrix and scree plot test, explaining 79.31% of total variance. Then, factor scores for all participants were obtained using the least squares regression approach, with mean and standard deviation equal to 0 and 1, respectively. Factor scores were considered as a measure of intensity of psychological problems. Thus, the higher scores indicate the higher intensity of psychological problems.

Table 3 presents association of MSDs patterns and psychological problems score in a multinomial logistic regression. Class 3; the class with low rate of complaints across all musculoskeletal sites, was deployed as the reference. It is obvious that odds ratios (ORs) indicate the odds of being in class 1 or class 2 compared to class 3 when psychological problems score increases one unit. In this regard, there was statistically significant association between patterns of MSDs and employees’ psychological problems score in both adjusted and unadjusted models. In a crude model (Model 1), increasing one standard deviation of psychological problems score significantly raises the chance of being in class 1, or equivalently the probability of musculoskeletal complaints in neck, shoulder, and joints increases for an individual (*p* value < 0.001). Moreover, there was significant association between psychological problems score and being in class 2 (increasing probability of musculoskeletal complaint in the back and upper back) (*p* value = 0.012). After adjustment for confounding variables including age, marriage status, doing exercise, smoking, work hours, job section, and body posture at work, only the association of complaints in class 1 with psychological problems score remained statistically significant (*p* value < 0.001). In addition, the association between the complaints in class 2 (lower and upper back) was not significant anymore (*p* value = 0.150).

**Table 3:** Association of latent class-derived patterns of MSD and psychological problems score

	Class 1	Class 2	Class 3^a^
	More complaints in neck, shoulder, and joints (*n* = 46)	More complaints in back, upper back (*n* = 137)	Low complaints in all musculoskeletal sites (*n* = 175)
	OR (95% CI)	OR (95% CI)	Reference
Model 1	1.96	1.35	–
	(1.41, 2.71)	(1.07, 1.72)	
Model 2	1.97	1.21	–
	(1.37, 2.81)	(0.93, 1.57)	

Model 1, unadjusted odds ratios in the multinomial logistic model. Model 2, adjusted for age, marital status, doing exercise, smoking, job section, work hours, and body posture at work. ^a^Class 3 as the reference category

**Fig. 2:**
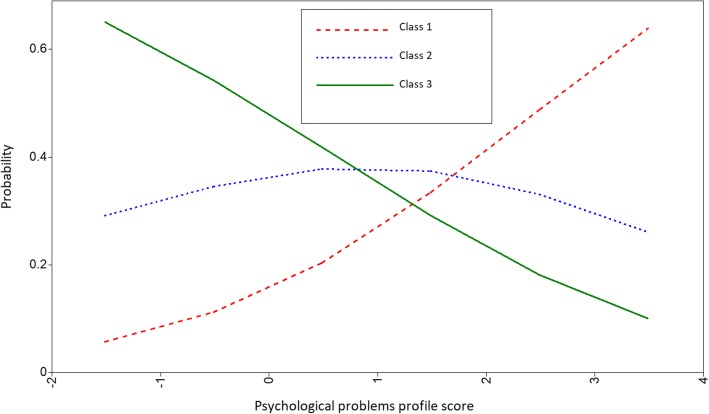
Probability of membership in identified classes of MSDs as a function of psychological problems scores. Class 1, high rate of MSDs in neck, shoulder, and joints (dashed line). Class 2, high rate of MSDs in upper and lower back (dotted line). Class 3, low MSDs prevalence in all sites (solid line)

The association between psychological problems score and the chance of being in each derived MSDs class, according to the adjusted model (Model 2) was depicted in Fig. 2. Increasing intensity of psychological problems is associated with a higher chance of musculoskeletal disorders loaded in class 1. In contrast, any change in intensity of psychological problems was not associated with the chance of being in class 2.

**Fig. 3:**
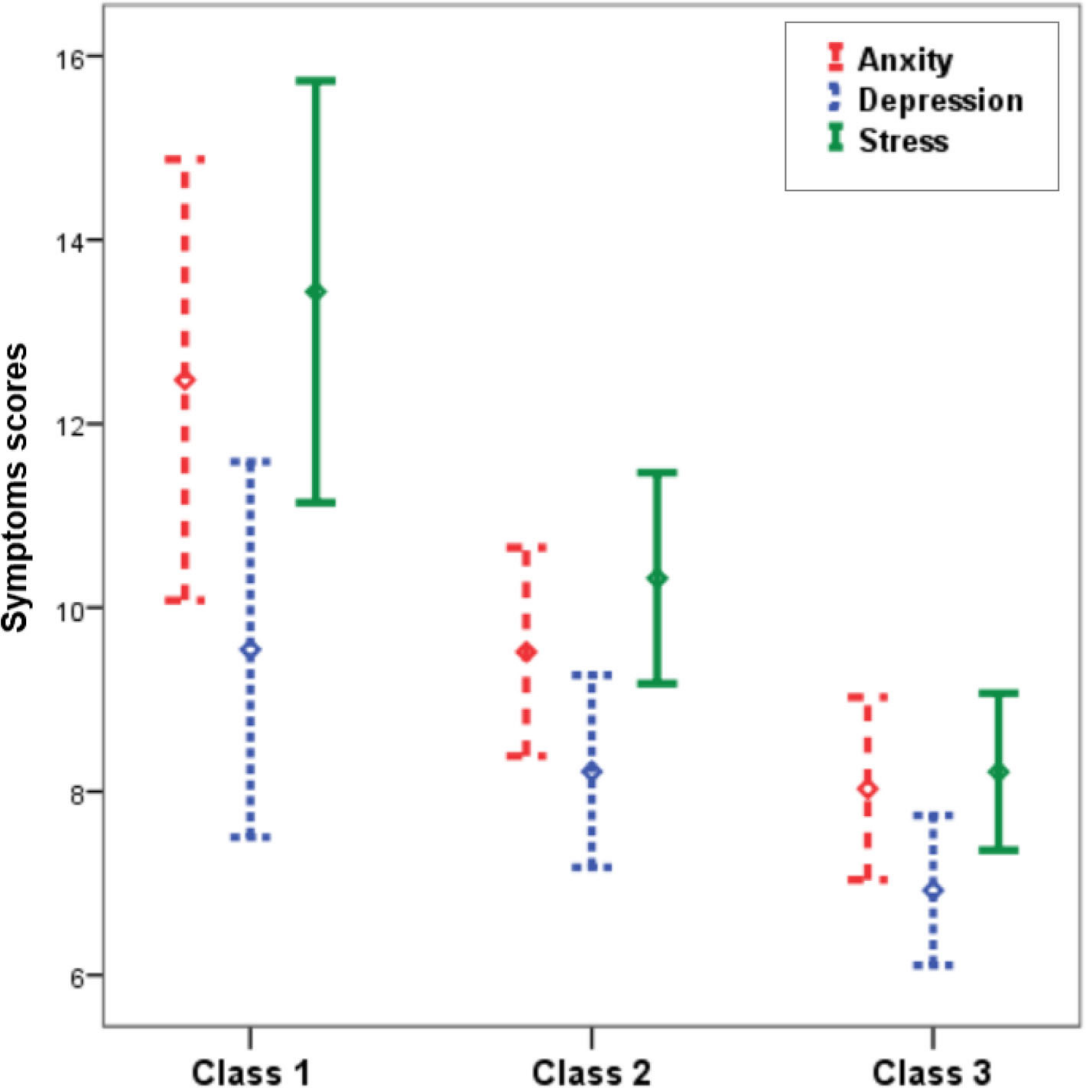
Mean and 95% confidence interval of anxiety (dashed line), depression (dotted line), and stress (solid line) scores across latent class-derived patterns of MSD. (Class 1, high rate of MSDs in neck, shoulder and joints; class 2: high rate of MSDs in upper and lower back; class 3, low rate of MSDs in all sites)

Figure 3 shows the distribution of depression, anxiety, and stress scores across the identified MSDS classes. All three measures had the highest mean in class 1 and the lowest was observed in class 3. There was a statistically significant difference between means of depression (*F* = 4.49, *p* value = 0.012), anxiety (*F* = 7.87, *p* value < 0.001), and stress scores (*F* = 12.62, *p* value < 0.001) across derived classes.

## Discussion

In the present study, we evaluated major patterns of MSDs and their association with psychological problems scores in a sample of industrial manufacturing employees. Based on the reported pain or complaints in nine musculoskeletal sites, three major classes were extracted using latent class analysis (LCA). Class 1 (12.9%) was characterized by a higher rate of complaints in joints and upper musculoskeletal sites, neck and shoulder, class 2 (38.2%) was recognized by a higher rate of complaints in the back and upper back, and class 3 (48.9%) was marked by a low rate of complaints in all musculoskeletal sites.

In the current study, because of the comorbidity of psychological problems [35], a combined measure was obtained from three common psychological problems including depression, anxiety, and stress (i.e., psychological problems score). There was a statistically significant association between the higher intensity of psychological problems and the increasing chance of MSDs in class 1, i.e., more complaints in the neck, shoulder, and joints.

Current study is the first to classify individuals based on their MSDs using LCA and evaluated psychological condition in the homogenous groups. Recent studies have emphasized on a multisite investigation of MSDs in the working and general population [6, 9]. In one study on 3710 French workers using a descriptive analysis of data, it was found out that two thirds of workers reported musculoskeletal symptoms in more than one anatomical site in the last year and the anatomical sites most frequently associated with other musculoskeletal symptoms were the neck, upper back, elbow, and hip [6]. In a study among Greek workers of different job groups, pain at multiple anatomical sites was reported common and the number of anatomical sites involving with musculoskeletal symptoms was suggested as a measure for describing severity of the impairment [9].

There are some studies that classified individuals to homogenous groups according musculoskeletal disorders through statistical approaches, e.g., clustering, structural equation mixture modeling (SEMM), and LCA [14–17]. Hartvigsen et al. investigated patterns of musculoskeletal pain based on a primary pain site in the 4817 adult Danes using latent class analysis [14]. In Gold et al.’s study among patients with upper extremity musculoskeletal disorders which employed cluster analysis, the patients were classified to mild through extrem muscculoskeletal disorders subgroups [16]. In a study on the Iranian general population, based on some neuro-skeletal indicators, including headache, back pain, pain in joints, eyesore, severe fatigue, dizziness and confusion, chills and extreme cold, and hot flashes in 3 months, the population was classified into two major subgroups using SEMM [17].

On the other hand, several systematic reviews have indicated the psychological problems that are risk factors of musculoskeletal symptoms [36, 37]. In a 10-year follow-up on metal industry workers, it was found out that musculoskeletal symptom scores were associated with change in the stress symptoms in men, as did the clinical findings in the neck-shoulder and low back regions [21]. Hear et al. using separate multinomial logistic regressions in a general-based study population showed that those with a current depressive disorder, remitted depressive, or anxiety disorder have high odds for musculoskeletal pain in at least one site of the back, neck, head, joints, or face [20]. Psychological distress in Remeet et al.’s study was found to be higher in patient care workers with musculoskeletal pain [22]. Depression and anxiety were identified as major determinants of neck pain [38]. Direct association of job stress and MSDs occurrence in at least on site was reported in different studies [39, 40]. In a study on computer operators, a significant association between job stress and musculoskeletal symptoms in the back was found but no significant association was obtained with MSDs in the neck, shoulders, elbows, hands, and wrists [41]. In a study among military personnel, significant association of anxiety and depression with MSDs was reported [42]. A systematic review integrated strong evidence of an association between knee pain and anxiety, depression, and poor mental health [43], including three randomized clinical trials that showed treatment with antidepressant drug was related to pain relief.

One question, not accurately settled in previous researches in this area, is which pattern of musculoskeletal symptoms will be most strongly associated with psychological disorders, particularly when adjusting for some important confounding variables such as body posture when working, demographics, and lifestyle-related variables. In evaluating association of psychological problems score with MSDs patterns, our findings implied MSDs in the neck, shoulder, and joints had significant direct association with psychological problems level in crud and adjusted models.

The link between psychological disorders and musculoskeletal disorders can be explained from the biological perspective [19]. Some investigators have reported the possible role of neurotransmitters [44] and cytokine receptors [45]. However, there is still no proved neurochemical explanation for the association of low mood and musculoskeletal condition [19]. The revealed evidence on pathogenesis of depression suggests that it is associated with dysfunction in the inflammatory cytokine production as a reaction to stressful events, dysregulation of autonomic nervous system [46, 47], and destabilizing impact on hypothalamic-pituitary-adrenal axis [48]. Each of these mechanisms also contributes to the provocation of chronic pain syndrome [46, 49]. Therefore, since the people involved with psychological problems are prone to inflammation, they are at a higher risk of developing joints dysfunction and pain.

It is important to mention some strengths and limitations of the present study. A major strength is the application of LCA for identifying a major pattern of MSDs, and stratifying study population based on it. Furthermore, the association of intensity of psychological problems with the identified classes considered a wide range of confounding variables. However, due to the cross-sectional nature of the study design, we could not infer cause–effect association from the findings. Because of low rate of female employees in the underlying population of the current study, the results were limited to males. Since there is evidence about sex disparities in MSDs prevalence [50, 51], further studies need to have a comprehensive view of MSDs patterns in both sex groups. Also, the data collected by self-reported questionnaires in the current study can lead to a misspecification of participants to the correct class.

## Conclusions

The results of the present study suggested that MSDs had a categorical structure and there was a considerable frequency and extent of multisite MSDs within the study population. Further research must be conducted in this field in order to provide a better comprehension of the characteristics and determinants of identified multisite MSDs. In addition, we showed that the employees’ psychological problems score composed of anxiety, depression, and stress were significantly associated with MSDs patterns. This finding might be useful for dealing with prevention and treatment programs. Finally, prevention and early detection of psychological symptoms need to be highlighted in workers’ mental healthcare programs since they impose a heavy burden on people and reduce productivity through consequent physical illnesses and disability.

## Abbreviations

AIC: Akaike’s information criteria
BIC: Bayes information criteria
CI: Confidence interval
DASS: Depression/Anxiety/Stress Scale
LCA: Latent class analysis
MSDs: Musculoskeletal disorders
NMQ: Nordic Musculoskeletal Questionnaire
OR: Odds ratio
